# Prevalence, Socio-Demographic Characteristics, and Co-Morbidities of Autism Spectrum Disorder in US Children: Insights from the 2020–2021 National Survey of Children’s Health

**DOI:** 10.3390/children12030297

**Published:** 2025-02-27

**Authors:** Mona Salehi, Sanobar Jaka, Aida Lotfi, Arham Ahmad, Mahdieh Saeidi, Sasidhar Gunturu

**Affiliations:** 1 Department of Psychiatry, BronxCare Health System, New York, NY 10456, USA; 2 Department of Psychiatry and Behavioral Sciences, University of Minnesota, Minneapolis, MN 55455, USA; 3 Department of Psychiatry, School of Medicine, Johns Hopkins University, Baltimore, MD 21287, USA; 4 Department of Psychiatry and Behavioral Sciences, Nassau University Medical Center, East Meadow, NY 11554, USA; 5 Department of Population Health, NYU School of Medicine, New York, NY 10016, USA; 6 School of Health and Welfare, Jönköping University, 551 11 Jönköping, Sweden; aida.lotfi@ju.se; 7 Department of Psychiatry, Icahn School of Medicine at Mount Sinai, New York, NY 10029, USA

**Keywords:** autism spectrum disorder, prevalence, adolescents, children, national survey of children’s health, severity

## Abstract

Background: The primary goal of our study is to assess the national US prevalence of autism spectrum disorder (ASD), along with its socio-demographic characteristics, severity, and co-occurring medical and psychiatric disorders, using data from the 2020–2021 National Survey of Children’s Health (NSCH). Methods: We analyzed 2020–2021 NSCH data to estimate the prevalence of ever-diagnosed and current ASD among 79,182 children and adolescents (3–17 years). Univariate and multivariate regression models were used to examine associations between medical and psychiatric co-morbidities, socio-demographic factors, and ASD severity. Results: Adolescents (11–17 years) and males were more likely to have ASD, with males comprising 78.7% of the ASD group. The mean age of the sample was 10.1 ± 4.6 years, and 3.2% had an ASD diagnosis. Children from lower-income households and those with caregivers who completed only a high school education were more likely to have ASD. Nearly 96.4% of children with ASD had at least one co-morbid condition. The most common neuropsychiatric co-morbidities were developmental delay (64%), behavioral and conduct problems (57.8%), and anxiety disorder (45.7%), while the most common medical conditions were allergies (32.4%), genetic disorders (26.2%), and asthma (12.6%). Gender disparities in ASD presentation were evident that females with ASD were more likely to experience vision problems, cerebral palsy, epilepsy, depression, and intellectual disability but had lower odds of ADHD and anxiety problems. Greater ASD severity was linked to higher odds of intellectual disability (OR: 5.8, *p* < 0.001), developmental delay (OR: 5.0, *p* < 0.001), epilepsy, Down syndrome (OR: 3.4, *p* < 0.001), vision problems (OR: 2.5, *p* < 0.001), and genetic disorders (OR: 2.3, *p* < 0.001). Conclusions: This study provides updated prevalence estimates of ASD and highlights the high burden of co-morbidities, emphasizing the need for comprehensive, multidisciplinary approaches in ASD management. Additionally, our findings emphasize gender differences in ASD presentation, which should be considered in future research and clinical practice to ensure more tailored diagnostic and intervention strategies.

## 1. Introduction

Autism Spectrum Disorder (ASD) is a neurodevelopmental disorder characterized by deficits in three main areas of functioning, including social interaction, communication skills, and pervasive or repetitive behaviors, as delineated in the DSM-5 [1]. Globally, autism prevalence is estimated at 0.72%, with higher rates observed in North America, high-income countries, and children aged 6–12 years [2]. Prevalence varies significantly across regions, ranging from 1 in 59 in the U.S. to 1 in 806 in Portugal [3].

A recent study reported that 1 in 36 children in the United States has ASD, with a male-to-female ratio of 4:1 [4]. ASD prevalence has been noted to be higher among youth from non-Hispanic white backgrounds compared to non-Hispanic black and Hispanic ethnic groups [5]. While diagnostic criteria for ASD can be met as early as 2 years of age, the average age for the first intervention has been reported to be after a child reaches 4 years of age. This is noteworthy because evidence suggests that parents might be capable of identifying concerns related to ASD in their children even before the child reaches 12 months of age [6]. These issues may arise from a shortage of experts, lengthy evaluation processes, high costs of care, and reluctance among healthcare providers to make referrals. Consequently, this results in delays and extended wait times for evaluations [7]. Socioeconomic factors, such as parents’ education and family income, play a significant role in how individuals with ASD experience life and receive the help they need. Families with lower incomes often face challenges when trying to access specialized therapies, education support, and healthcare services, and this can affect their long-term outcomes [5].

ASD is categorized into three severity levels based on the DSM-5. Level 1 requires some support; Level 2 needs substantial support, and Level 3 requires very substantial support. This classification helps tailor interventions to individuals’ unique needs within the autism spectrum [1]. These severity levels can be assigned by clinicians using clinical observations and psychological evaluations to judge an individual’s specific deficits in various domains, indicating the level of support required [8]. Due to its highly variable and complex genetic nature, ASD exists within a web of interrelated factors, causing individuals with ASD to often contend with a constellation of co-morbidities [9]. From genetic to psychiatric to medical, some common co-morbid conditions individuals with ASD can be implicated with are Fragile X syndrome, Rett Syndrome, attention deficit hyperactivity disorder (ADHD), depression, anxiety, intellectual disability, schizophrenia, epilepsy, gastrointestinal disturbances, and susceptibility to infections [10,11,12,13]. Each co-morbidity is coupled with its own level of increased overall dysfunction, closely affecting the severity of ASD and causing significant clinical impairment and additional disease burden [8]. Targeted interventions for co-morbid conditions improve functioning more than nonspecific treatments. EEG neurofeedback, including a 12-week Mente Autism course, enhances brain activity, behavior, and social skills in ASD, with effects lasting up to 12 months [14,15,16].

In this nationally representative study, we aim to estimate the parent-reported prevalence of ASD among children and adolescents in the United States and its association with family income and parental education levels. Additionally, we will examine medical and psychiatric co-morbidities, their correlation with severity levels, and the socio-demographic characteristics of affected individuals, including age groups (3–5, 6–10, and 11–17 years), gender, and ethnicity.

## 2. Materials and Methods

### 2.1. Study Design

This study examines the characteristics and co-morbid conditions of ASD in individuals aged 3 to 17 years, utilizing data from The National Survey of Children’s Health (NSCH) for the years 2020–2021 in the United States. The NSCH is a comprehensive, nationally representative survey conducted by the Health Resources and Services Administration’s Maternal and Child Health Bureau (HRSA MCHB) within the U.S. Department of Health and Human Services [17]. Its primary purpose is to evaluate the well-being, access to quality health care, familial neighborhood and education, and social contexts of youth residing in the United States, along with associated influencing factors.

### 2.2. Procedure and Sampling in the Dataset

The NSCH used 42,777 surveys in 2020 (response rate: 42.4%) and 50,892 surveys in 2021 (response rate: 40.3%) [17]. The analytic sample of this study included 79,182 children and adolescents aged between 3 to 17 years. An address-based sample was selected from an extract of the Census Bureau’s Master Address File (MAF), which covered 50 states and the District of Columbia. The sample frame employed flags based on administrative records to establish four distinct and non-overlapping strata [17].

The survey was administered online and via mail. Randomly selected households received instructions to access the survey online, with some also receiving a paper version. Survey questions are summarized in Supplemental Table S1. The data collection involved a two-phase methodology, starting with a household screener and followed by a comprehensive questionnaire for a selected youth’s parents or caregivers. Various strategies were employed to enhance response rates, including clear question phrasing, multiple response mode options, cash incentives, and other interventions.

Data from the NSCH 2020–2021 underwent a weighting process involving adjustments for nonresponse, post-stratification, and raking. The raking adjustment iteratively fits case weights across dimensions such as state-by-household poverty ratio, respondent’s education, selected children’s race/ethnicity, age group, and nationally selected children’s race/ethnicity and sex. This process involved up to 100 iterations or until the weights converged to population totals [17]. Further details on the selection and sampling methodology can be found on the DRC website at childhealthdata.org.

### 2.3. Confidentiality

Participation in the 2021 NSCH was voluntary, and all data collected that could potentially identify an individual person are confidential. Data are kept private in accordance with applicable law. Respondents are assured of the confidentiality of their replies in accordance with 13 U.S.C. Section 9. All access to Title 13 data from this survey is restricted to Census Bureau employees and those holding Census Bureau Special Sworn Status pursuant to 13 U.S.C. Section 23(c). In compliance with this law, all data released to the public are only in a statistical format. No information that could personally identify a respondent or household may be released. The Screener and Topical public use data files went through a thorough disclosure review process and were approved by the Census Disclosure Review Board prior to release.

### 2.4. Data Variables and Measures

#### 2.4.1. Dependent Variables

This analysis considered various independent variables, encompassing socio-demographic factors such as age, race, ethnicity, and family income, classified based on the federal poverty level and the highest education of adults in the household. The primary dependent variable of interest was the presence or absence of ASD, as measured by the report of parents or caregivers. They were asked, “Does this child currently have autism or ASD, including Asperger’s disorder and pervasive developmental disorder?” The secondary dependent variable was the severity of ASD, which was measured by asking the parents or caregivers, “Would you describe this child’s current autism or autism spectrum disorder as mild, moderate, or severe?” (Supplemental Table S1).

#### 2.4.2. Independent Variables

Co-morbid conditions, including psychiatric disorders such as ADHD, Tourette syndrome, depression, and anxiety problems, as well as medical issues, including heart disorders, developmental delay, intellectual disability, behavioral and conduct problems, asthma, allergies, arthritis, cerebral palsy, diabetes, down syndrome, epilepsy, frequent/severe headaches, hearing problems, genetic disorders, and vision problems, were included in this analysis.

### 2.5. Statistical Analysis

All statistical analyses were conducted using Stata version 17.0. Continuous variables were presented as mean ± standard deviation, and categorical variables were presented as frequency (percentage). Initial comparisons between the two groups (children with and without ASD) for continuous variables were performed using *t*-tests. Univariate and multivariate regression models were utilized to examine the association between medical and psychiatric co-morbidities as well as socio-demographic factors and ASD. In the univariate models, each covariate’s association with ASD was assessed independently. The multivariate models evaluated the association between each covariate and ASD while adjusting for all other covariates. Adjusted odds ratios (ORs) and 95% confidence intervals (CIs) were generated by the regression models, indicating the increased odds of ASD associated with each covariate after controlling for other variables.

## 3. Results

The NSCH dataset for 2020–2021 included 93,669 participants, covering children and adolescents aged 0 to 17 years. Of these, 79,182 individuals aged 3 to 17 years were surveyed regarding their ASD diagnosis and were the focus of this study. The participants’ average age was 10.1 ± 4.6 years (mean ± SD). Notably, 2568 individuals, or 3.2%, had a confirmed current diagnosis of ASD (Table 1). Adolescents (11–17 years) had the highest prevalence of ASD at 54.7%. A significant difference was noted in the sex distribution, with males accounting for 78.7% of children with ASD compared to 51.0% in the non-ASD group. Conversely, females constituted only 21.3% of the ASD group, compared to 49.0% in the non-ASD group. The distribution of race/ethnicity was relatively similar between ASD and non-ASD groups. However, there was a slightly higher prevalence of multi-race children in the ASD group (8.3% vs. 7.1%). Children with ASD were more likely to come from lower-income households, with 17.3% living below the poverty level compared to 12.5% in the non-ASD group. Only 30.1% of children with ASD were from households with incomes ≥400% of the federal poverty level. Households of children with ASD had a higher prevalence of adults with only a high school degree (16.6% vs. 13.5% in non-ASD households), as shown in Table 1.

Adolescents (11–17 years) had significantly higher odds of having ASD (OR: 1.5, *p* < 0.001). Children in the school-age group (6–10 years) also showed increased odds of ASD (OR: 1.3, *p* < 0.001). Female children had significantly lower odds of having ASD compared to males (OR: 0.3, *p* < 0.001). Asian children had lower odds of ASD (OR: 0.7, *p* < 0.05). Multi-race children had higher odds of ASD (OR: 1.2, *p* < 0.05). Children from higher-income households (≥400% of the federal poverty level) had significantly lower odds of ASD (OR: 0.5, *p* < 0.001). Households with incomes between 200% and 399% of the federal poverty level also had lower odds of ASD (OR: 0.7, *p* < 0.001). Children from households where adults had a high school degree had higher odds of ASD (OR: 1.5, *p* < 0.01). Similarly, households with more than a high school education had increased odds of ASD (OR: 1.5, *p* < 0.01), as shown in Table 2.

**Figure 1:**
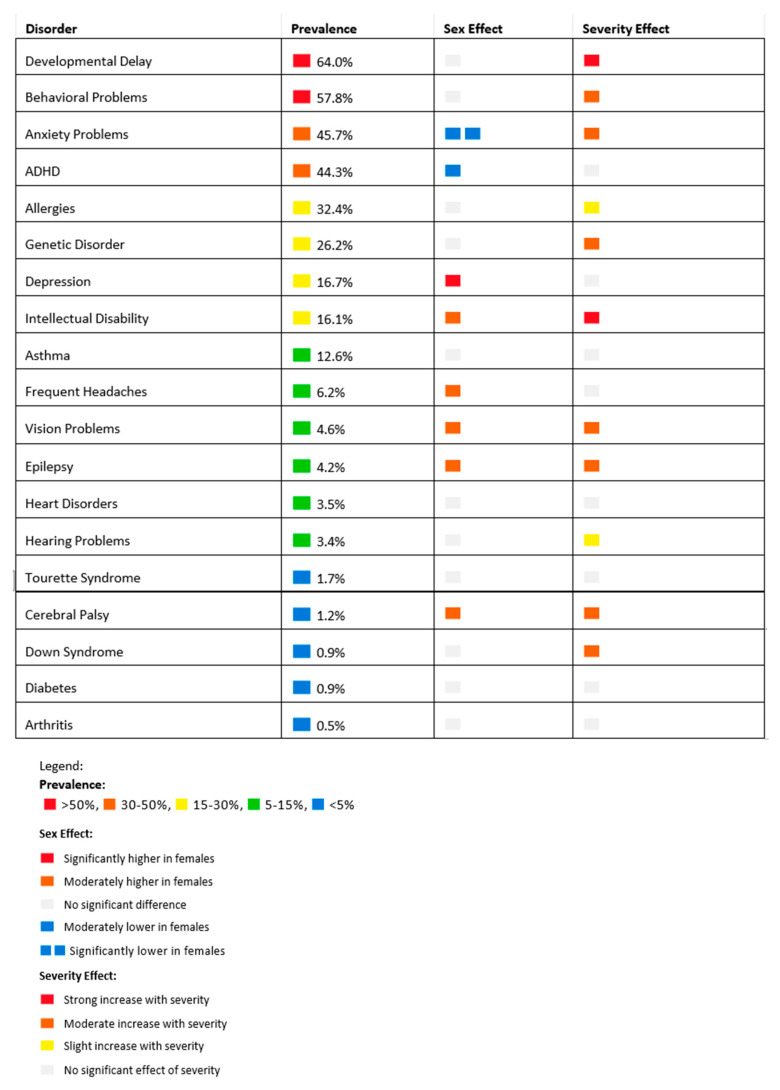
Co-morbidities in Autism Spectrum Disorder (ASD)—Heat Map Visualization.

The most common neuropsychiatric co-morbidity was developmental delay (64%). Behavioral and conduct problems (57.8%) and anxiety problems (45.7%) were also prevalent. Allergies (32.4%) were the most common medical co-morbidity. Genetic disorders (26.2%) and asthma (12.6%) followed. Females with ASD had significantly higher odds of co-morbid vision problems (OR: 2.3, *p* < 0.001), cerebral palsy (OR: 2.2, *p* = 0.042), frequent/severe headaches (OR: 1.7, *p* = 0.002), epilepsy (OR: 1.7, *p* = 0.014), depression (OR: 1.6, *p* < 0.001), and intellectual disability (OR: 1.5, *p* = 0.001). Conversely, females had lower odds of ADHD (OR: 0.8, *p* = 0.007) and anxiety problems (OR: 0.35, *p* < 0.001). More severe ASD was associated with higher odds of intellectual disability (OR: 5.8, *p* < 0.001), developmental delay (OR: 5.0, *p* < 0.001), epilepsy (OR: 3.4, *p* < 0.001), Down syndrome (OR: 3.0, *p* = 0.01), vision problems (OR: 2.5, *p* < 0.001), behavioral and conduct problems (OR: 2.4, *p* < 0.001), genetic disorders (OR: 2.3, *p* < 0.001), hearing problems (OR: 1.6, *p* = 0.03), and anxiety problems (OR: 1.3, *p* < 0.001), as shown in Table 3 and Figure 1.

## 4. Discussion

### 4.1. Prevalence of ASD

The parent-reported prevalence of ASD in U.S. children and adolescents was 3.2% in this study. This finding represents a slight increase from the previous report of 3.14%, according to the 2019–2020 NSCH survey [18]. The Autism and Developmental Disabilities Monitoring (ADDM) Network reported a prevalence of 2.3% in 2018, while the NSCH in 2016 reported a prevalence of 2.5% [19,20]. However, it is important to interpret these findings with caution, as the estimates are derived from different systems that reflect different ages and populations: children aged 8 years from 11 local populations in the ADDM versus children aged 3–17 years from populations across the entire USA in the NSCH [19,20]. Additionally, the variations in ASD prevalence may be attributed to the inherent nature of ASD as a spectrum disorder characterized by diverse traits that can influence the definitions and diagnostic criteria of ASD [1].

### 4.2. Socio-Demographic Associations

#### 4.2.1. Age-Groups

Our analysis revealed a significantly higher prevalence of ASD in the adolescent (11–17 years) age group, which aligns with the existing literature indicating that ASD diagnoses become more stable and reliable with age [21]. A longitudinal study by Lord et al. found that diagnostic stability increased from 84% at age 2 to 94% at age 9 [21]. This improved accuracy in older children and adolescents is likely due to the more pronounced manifestation of ASD symptoms over time and the availability of longer developmental histories [22,23].

The higher prevalence of ASD in older children may be influenced by increased screening opportunities within school settings, where structured assessments and teacher observations facilitate the identification of traits that may have previously gone unnoticed [24]. Other contributing factors include improved diagnostic accuracy, greater awareness, evolving diagnostic criteria, and the rising social demands of adolescence, which make autistic traits more apparent [25,26]. Additionally, the mean age of ASD diagnosis remains over four years, as some children exhibit subtle symptoms early in life or follow a prolonged trajectory of symptom development, leading to delayed recognition and diagnosis. Socioeconomic disparities and limited access to specialized care further contribute to delays in early childhood identification, resulting in a higher proportion of diagnoses occurring later [27].

Beyond these individual-level factors, system-level inefficiencies also contribute to diagnostic delays. High rates of non-attendance, inappropriate referrals, workflow inefficiencies, and the absence of clear care pathways create barriers in the diagnostic process [28].

#### 4.2.2. Gender Differences

We observed a male-to-female ratio of approximately 3.7:1 in the prevalence of ASD. This finding is consistent with the well-established gender disparity in ASD diagnoses reported in numerous studies. Recent research continues to support a significant male predominance in ASD, though the exact ratios vary across different populations and age groups [29,30,31]. The prevailing belief that ASD is more frequently identified in males compared to females has spurred the development of several hypotheses that aim to explain the characteristics and causes of ASD. These ideas include the extreme male brain theory [32], the female protective effect theory [22,33,34], and the female autism phenotype theory [35,36,37]. The extreme male brain theory suggests that understanding gender differences involves considering “empathizing” and “systemizing”. Evidence indicates that males tend to excel in systematization, while females have distinct cognitive traits. This theory proposes that ASD might represent an extreme male cognitive profile [32]. The “female protective model/effect” stems from the consistent male predominance in ASD. It assumes that ASD risk is spread throughout the population and suggests that females have a protective factor against autism (34). Due to this protective effect, ASD affects women less often than men. High-functioning ASD has a male-to-female ratio of 7:1, while moderate to severe Intellectual Disability has a ratio of 2:1, indicating female protection even in the presence of risk factors [22,34,38]. Notably, the gender ratio in ASD appears to be influenced by cognitive ability. A large-scale surveillance study reported a male-to-female ratio of 4.3:1 in children with ASD without intellectual disability, compared to a ratio of 3.2:1 in those with co-occurring intellectual disability [31]. Recent studies have also highlighted that the true gender ratio in ASD may be lower than previously thought. Research suggests that many autistic females may be missed by current diagnostic criteria, potentially due to a female autism phenotype that differs from the traditionally recognized male presentation [29]. The female autism phenotype theories suggest a unique expression of autism in females, but current autism research is male-centric [39,40,41]. A comprehensive review analyzed 54 studies and found a mean male-to-female ratio of 3.25:1 in ASD diagnoses, which closely aligns with our observed ratio. However, the authors suggest that this disparity may be influenced by underdiagnosis or misdiagnosis of females with ASD [30].

#### 4.2.3. Other Socio-Demographic Factors

We found the highest odds of ASD in multi-race youth, as well as youth with high school and higher than high school caregiver educational level. There are some differences between our study findings and the Centers for Disease Control and Prevention (CDC) report of racial differences in youth with ASD, as they found the highest prevalence of ASD in youth with Asian/pacific islander descents [42], which could be the result of different methodologies and instruments. Research has shown that the prevalence of ASD was initially higher in white children compared to black or Hispanic children but gradually equalized by 2016 and 2018 [19,42]. Recent reports in 2020 marked for the first time that ASD rates were lower in white children than in other groups at age 8, and a similar trend was observed among 4-year-olds in 2018 [19,42]. This shift may be due to better screening, increased awareness, and improved services for historically marginalized populations [42]. Youth with caregivers having at least a high school education are more likely to have ASD, a finding consistent with other studies [42,43]. Diagnostic odds ratios for parental education and income are complex. Families with lower educational attainment have a reduced likelihood of receiving an ASD diagnosis, potentially linked to inequities in healthcare access and outcomes. Lower-educated families may face limited healthcare services, decreasing their chances of obtaining a medical diagnosis for their child [42].

We found that ASD in children and adolescents is higher among families with less than 100% FPL income. The ADDM Network Reported that the prevalence of ASD in 2020 exhibited a correlation with lower socioeconomic status [44]. Although some previous studies reported contrasting findings [5], these findings offer further evidence of improved ASD identification for youth regardless of their socioeconomic level. As research continues to grow on better identification methods, attention may shift toward understanding the factors, such as socioeconomic determinants of health, that could lead to higher rates of detected impairment in specific communities [44].

### 4.3. Co-Morbid Conditions and Severity of ASD

Our study revealed that over 96% of individuals with ASD had at least one co-morbid condition. The most prevalent co-morbidities we identified were developmental delay, behavioral and conduct problems, anxiety disorders, allergies, genetic disorders, and asthma. This high rate of co-occurring conditions aligns with and extends upon previous research in this field. A large-scale study also found that 70.8% of individuals with ASD had at least one co-occurring psychiatric condition. The most common co-morbidities they identified were anxiety disorders (39.6%), ADHD (28.1%), and depressive disorders (23.1%) [45]. Children with ASD have high rates of anxiety, partly due to sensory hyper-reactivity. Overwhelming stimuli like noise and light can trigger avoidance behaviors, which, over time, reinforce anxiety and make adaptation more challenging [46].

These findings are consistent with earlier research in which 70% of children with ASD had at least one co-morbid psychiatric disorder, with social anxiety disorder, ADHD, and oppositional defiant disorder being the most prevalent [47]. This analysis highlights the persistently high rates of psychiatric co-morbidities in ASD across different studies and populations.

Regarding medical co-morbidities, our findings of high rates of allergies and asthma are supported by the recent literature. A study by Xu et al. (2018) found that children with ASD had significantly higher rates of allergic diseases, including asthma (OR: 1.51), allergic rhinitis (OR: 1.65), and atopic dermatitis (OR: 1.38) compared to neurotypical children [48]. Gastrointestinal (GI) issues continue to be recognized as a significant co-morbidity in ASD. Children with ASD were 2.6 to 4.2 times more likely to experience GI symptoms compared to children without ASD, as per a recent review [49]. Common GI issues included constipation, diarrhea, and abdominal pain [49]. Research suggests that the Nlgn3 R451C mutation, associated with autism, reduces Nlgn3 mRNA expression in cholinergic enteric neurons, potentially disrupting gut–brain communication and contributing to gastrointestinal disturbances in ASD [50,51]. Developing interventions, such as dietary choline supplementation and Probiotic/colostrum supplementation, may further modulate cholinergic signaling, potentially improving cognitive function and alleviating GI symptoms in children with ASD [52,53,54].

Over 96% of ASD patients had at least one co-morbid condition. We found the most common co-morbid conditions with ASD to be developmental delays, behavioral and conduct problems, anxiety problems, allergies, genetic disorders, and asthma. Simonof et al. [47] found that 70% of children with ASD in a population-derived cohort had at least one co-morbid psychiatric disorder. They found the most common co-occurring psychiatric disorders with ASD to be social anxiety disorder, ADHD, and oppositional defiant disorder [47]. Similarly, Leyfer et al. found the most prevalent co-morbid psychiatric conditions in autistic children to be specific phobia, obsessive-compulsive disorder (OCD), and ADHD, respectively [14]. Regarding medical co-morbidities, the previous literature reported higher rates of allergies, including skin allergies, food allergies, asthma, as well as gastrointestinal problems [55]. These findings suggest that individuals with ASD face extra challenges when dealing with accompanying co-morbid conditions, which is clinically important as earlier screening for co-morbidity can aid in establishing additional priorities for assessment and more effective treatment options [56].

This study utilizes data from the National Survey of Children’s Health (NSCH) for 2020–2021, a comprehensive, nationally representative survey. The analytic sample included 79,182 children and adolescents aged 3 to 17 years, covering all 50 states and the District of Columbia for broad geographical representation. It employs a sophisticated sampling strategy, including an address-based sample from the Census Bureau’s Master Address File (MAF) and administrative records to establish four distinct strata. The survey was administered both online and via mail, enhancing accessibility and response rates.

This study examines a wide range of medical and psychiatric co-morbidities associated with ASD and analyzes ASD severity levels, providing insights into the relationship between severity and various factors. These strengths contribute to the study’s reliability and generalizability to the broader U.S. population of children and adolescents with ASD. This methodology provides important insights into autism spectrum disorder and can serve as a valuable model for other countries, particularly those that lack large-scale, standardized data collection on developmental disorders. Expanding such survey-based approaches globally would improve ASD prevalence estimates and enhance early detection efforts. Making these data accessible to researchers worldwide can help bridge knowledge gaps and support international public health strategies aimed at better ASD management and policy planning.

However, this study has several limitations. It relies on parent-reported data for ASD diagnosis and severity, which may introduce reporting bias. Additionally, as a cross-sectional survey, it provides a snapshot at a specific point in time, limiting the ability to establish causal relationships or track longitudinal changes. The NSCH response rates—42.4% in 2020 and 40.3% in 2021—may contribute to nonresponse bias, potentially affecting the representativeness of the findings. Recalling bias is another concern, particularly for parents reporting on older children’s past diagnoses and symptoms. Furthermore, this study focuses on children and adolescents aged 3–17 years, potentially overlooking early diagnoses in younger children or cases of ASD diagnosed in adulthood. Despite efforts to ensure a representative sample, certain populations may be underrepresented due to language barriers or limited access to the survey.

Another key limitation is that ASD severity was based on parental subjective assessment (mild, moderate, or severe), which may not align with standardized clinical classifications. Similarly, parent-reported co-morbidities may not accurately reflect the full clinical picture, as caregiver perspectives can be influenced by personal experiences, levels of stress, and access to professional guidance. Research suggests that family involvement in ASD interventions significantly shapes parental perceptions and reporting of symptoms [43]. In Spain, the Family-Centered Practices Scale (FCPS) has been validated to assess the integration of family participation in early intervention, highlighting the influence of caregiver experiences on ASD-related outcomes [57]. These findings indicate that variations in parental stress, cultural perspectives on ASD, and access to professional support may contribute to inconsistencies in parent-reported data. To improve diagnostic accuracy and minimize bias, future research should incorporate standardized clinician-administered assessments alongside parental reports.

Moreover, the cross-sectional design prevents tracking long-term trends in ASD prevalence, severity, and co-morbidities. While this study accounts for multiple socio-demographic factors, unmeasured confounding variables may still influence the results. Additionally, while pharmacological treatments play a role in managing ASD-related symptoms, their efficacy and risks were not within the scope of this study. Future research should explore both pharmacological and non-pharmacological treatment approaches to provide a more comprehensive understanding of ASD management. Future studies should conduct comparative analyses of ASD prevalence across countries and investigate the underlying risk factors contributing to these disparities.

## 5. Conclusions

Our findings suggest that male adolescents, multi-race individuals, and those with higher-educated families, as well as lower family income levels, may have increased odds of ASD, while females, higher-income households, and Asian races may have lower odds. More than 90% of children with ASD had at least one co-morbid condition, particularly developmental delay and behavioral issues, which can affect the severity of ASD. Identifying and addressing these co-morbidities early is crucial for clinical management and support.

## Figures and Tables

**Table 1:** Socio-demographic characteristics of children with and without autism spectrum disorder.

Socio-Demographic Characteristics	Total (n = 79,182)	ASD (n = 2568)	No ASD (n = 76,614)
Mean Age Years (SD)	10.1 ± 4.6	10.7 ± 4.4	10.0 ± 4.6
Age Groups (%)			
Preschool (3–5)	23.8	17.6	23.6
School (6–10)	28.1	27.6	28.1
Adolescents (11–17)	48.1	54.7	48.3
Sex (%)			
Male	51.9	78.7	51
Female	48.1	21.3	49
Race (%)			
Hispanic	13.5	14.3	13.5
White	65.8	65.7	65.9
Black	6.8	6.9	6.8
Asian	5.7	4.1	5.7
American Indian/Alaska Native	0.7	0.6	0.7
Native Hawaiian/Other Pacific Islander	0.3	0.2	0.3
Multi-Race	7.1	8.3	7.1
Federal Poverty Level (%)			
≥400%	40.3	30.1	40.6
200–399%	30.4	31.1	30.4
100–199%	16.7	21.6	16.5
<100%	12.6	17.3	12.5
Highest Education of Adults in Household (%)			
Less than high school	2.7	2.5	2.7
High school degree	13.5	16.6	13.5
More than high school	83.7	80.9	83.8

**Table 2:** Univariate and Multivariate Analysis of Socio-Demographic Factors Associated with Autism Spectrum Disorder.

Socio-Demographic Characteristics	Univariate Analysis OR (95% CI)	Univariate Analysis *p*-Value	Multivariate Analysis OR (95% CI)	Multivariate Analysis *p*-Value
Age Groups				
Preschool: 3–5	Reference	<0.001	Reference	<0.001
School: 6–10	1.3 (1.2–1.5)		1.3 (1.1–1.5)	
Adolescents: 11–17	1.5 (1.4–1.7)		1.5 (1.4–1.7)	
Sex				
Male	Reference	<0.001	Reference	<0.001
Female	0.3 (0.2–0.3)		0.3 (0.2–0.3)	
Race				
Hispanic	Reference	<0.001	Reference	<0.05
White	0.9 (0.8–1.1)		1.04 (0.9–1.2)	
Black	0.9 (0.8–1.2)		0.9 (0.7–1.1)	
Asian	0.7 (0.5–0.8)		0.7 (0.6–0.9)	
American Indian/Alaska Native	0.8 (0.5–1.4)		0.8 (0.4–1.3)	
Native Hawaiian/Other Pacific Islander	0.6 (0.2–1.5)		0.6 (0.2–1.4)	
Multi-Race	1.1 (0.9–1.3)		1.2 (1.0–1.4)	
Federal Poverty Level				
<100%	Reference	<0.001	Reference	<0.001
100–199%	0.9 (0.8–1.1)		0.9 (0.8–1.0)	
200–399%	0.7 (0.6–0.8)		0.7 (0.6–0.8)	
≥400%	0.5 (0.5–0.6)		0.5 (0.4–0.6)	
Highest education of adults in the household				
Less than high school	Reference	<0.05	Reference	<0.01
High school degree	1.4 (1.1–1.8)		1.5 (1.1–2)	
More than high school	1.1 (0.8–1.4)		1.5 (1.1–1.9)	

**Table 3:** Prevalence of co-morbid disorders in children with ASD (n = 2568), by sex and ASD severity.

Disorders	ASD (n = 2568)	Sex OR (95% CI)	Sex *p*-Value	ASD Severity OR (95% CI)	Severity *p*-Value
Attention Deficit Hyperactivity Disorder (ADHD)	44.3%	M: Reference	*p*-Value: 0.000	1: Reference	*p*-Value: 0.708
		F: 0.8 (0.6–0.9)		2: 1.03 (0.9–1.2)	
Tourette Syndrome	1.7%	M: Reference	*p*-Value: 0.000	1: Reference	*p*-Value: 0.062
		F: 0.6 (0.2–1.4)		2: 1.8 (0.9–3.5)	
Depression	16.7%	M: Reference	*p*-Value: 0.000	1: Reference	*p*-Value: 0.650
		F: 1.6 (1.3–2.0)		2: 0.9 (0.2–1.2)	
Anxiety Problems	45.7%	M: Reference	*p*-Value: 0.000	1: Reference	*p*-Value: 0.000
		F: 0.35 (0.3–0.4)		2: 1.3 (1.3–1.8)	
Heart Disorders	3.5%	M: Reference	*p*-Value: 0.000	1: Reference	*p*-Value: 0.953
		F: 1.3 (0.8–2.1)		2: 0.9 (0.6–1.5)	
Developmental Delay	64.0%	M: Reference	*p*-Value: 0.000	1: Reference	*p*-Value: 0.000
		F: 1.02 (0.8–1.2)		2: 5.0 (4.1–5.9)	
Intellectual Disability	16.1%	M: Reference	*p*-Value: 0.000	1: Reference	*p*-Value: 0.000
		F: 1.5 (1.2–1.9)		2: 5.8 (4.4–7.7)	
Behavioral and Conduct Problems	57.8%	M: Reference	*p*-Value: 0.000	1: Reference	*p*-Value: 0.000
		F: 0.9 (0.7–1.0)		2: 2.4 (2.0–2.8)	
Asthma	12.6%	M: Reference	*p*-Value: 0.000	1: Reference	*p*-Value: 0.365
		F: 1.06 (0.8–1.4)		2: 1.1 (0.9–1.4)	
Allergies	32.4%	M: Reference	*p*-Value: 0.000	1: Reference	*p*-Value: 0.006
		F: 0.8 (0.7–1.03)		2: 1.3 (1.06–1.5)	
Arthritis	0.5%	M: Reference	*p*-Value: 0.001	1: Reference	*p*-Value: 0.382
		F: 0.6 (0.1–2.7)		2: 1.6 (0.5–4.8)	
Cerebral Palsy	1.2%	M: Reference	*p*-Value: 0.000	1: Reference	*p*-Value: 0.008
		F: 2.2 (1.02–4.6)		2: 3 (1.3–7.0)	
Diabetes	0.9%	M: Reference	*p*-Value: 0.000	1: Reference	*p*-Value: 0.954
		F: 1.2 (0.5–2.9)		2: 0.9 (0.4–2.1)	
Down Syndrome	0.9%	M: Reference	*p*-Value: 0.000	1: Reference	*p*-Value: 0.002
		F: 2.1 (0.9–4.7)		2: 4.5 (1.5–13.4)	
Epilepsy	4.2%	M: Reference	*p*-Value: 0.000	1: Reference	*p*-Value: 0.000
		F: 1.7 (1.1–2.6)		2: 3.4 (2.1–5.5)	
Frequent/Severe Headaches	6.2%	M: Reference	*p*-Value: 0.000	1: Reference	*p*-Value: 0.838
		F: 1.7 (1.2–2.4)		2: 1.06 (0.7–1.5)	
Hearing Problems	3.4%	M: Reference	*p*-Value: 0.000	1: Reference	*p*-Value: 0.032
		F: 1.2 (0.7–1.9)		2: 1.6 (1.03–2.5)	
Genetic Disorder	26.2%	M: Reference	*p*-Value: 0.000	1: Reference	*p*-Value: 0.000
		F: 1.2 (0.9–1.5)		2: 2.3 (1.9–2.8)	
Vision Problems	4.6%	M:Reference	*p*-Value: 0.000	1: Reference	*p*-Value: 0.000
		F: 2.3 (1.6–3.5)		2: 2.5 (1.6–3.8)	

## Data Availability

The dataset utilized in this study is publicly available from the Data Resource Center for Child and Adolescent Health website: https://www.childhealthdata.org/learn-about-the-nsch/NSCH, accessed on 1 January 2025.

## Supplementary Materials

The following supporting information can be downloaded at https://www.mdpi.com/article/10.3390/children12030297/s1, Table S1: National Survey of Children’s Health Questions.
